# Primary angiosarcoma of the nasal septum: a rare case report and literature review

**DOI:** 10.1017/S0022215126104678

**Published:** 2026-08

**Authors:** Yun Jin Kang, Min Su Kim, Youn Sun Park, Jin-Hee Cho

**Affiliations:** Department of Otorhinolaryngology-Head and Neck Surgery, Yeouido St. Mary’s Hospital, College of Medicine, The Catholic University of Korea, Seoul, Korea

**Keywords:** angiosarcoma, nasal septum, radiotherapy, sarcoma

## Abstract

**Objectives:**

Angiosarcoma is a rare malignant vascular tumour. Its occurrence in the nasal cavity is uncommon, and septal cases are particularly rare. Because symptoms are often non-specific, nasal septal angiosarcoma may initially mimic benign vascular lesion and delay diagnosis.

**Methods:**

We report a 46-year-old woman with recurrent epistaxis and nasal obstruction. Nasal endoscopy and excisional biopsy were performed, with histopathology and immunohistochemistry.

**Results:**

Endoscopy revealed a 1.0 × 0.5 cm mass on the anterior septum. The lesion appeared completely excised on gross inspection; however, histopathology demonstrated infiltrative margins with residual tumour at the resection edges. Microscopy showed irregular vascular channels, pleomorphic endothelial cells, high mitotic activity and focal necrosis. Immunohistochemistry confirmed endothelial differentiation. No cartilage invasion was identified on histology.

**Conclusion:**

This case highlights the diagnostic challenge of nasal septal angiosarcoma and the emphasises the importance of early biopsy, histopathological confirmation and multidisciplinary management.

## Introduction

Angiosarcoma is a rare and highly aggressive malignant tumour of endothelial origin, accounting for less than 2 per cent of all soft tissue sarcomas.^1^ It predominantly arises in the skin and soft tissues of the head and neck region, particularly in the scalp and face of elderly individuals. However, its presence in the nasal cavity and paranasal sinuses is exceedingly rare, with only a few cases documented worldwide.

Among reported sinonasal angiosarcomas, primary involvement of the nasal septum is particularly uncommon. Previous cases have primarily described angiosarcomas originating from the nasal cavity, maxillary sinus and ethmoid sinus, often exhibiting extensive local invasion and poor prognosis.^2^^–^^4^ The management of sinonasal angiosarcoma is complicated by the lack of diagnostic criteria and standardised treatment guidelines. This report reviews previously documented cases to understand their clinical presentation, imaging characteristics, histopathological features, treatment approaches and patient outcomes. The analysis highlights the unique aspects of the case, emphasising its distinct clinical course and limited local involvement.

Due to its non-specific symptoms, including recurrent epistaxis and nasal obstruction, nasal septal angiosarcoma is frequently misdiagnosed as a benign vascular lesion such as haemangioma, pyogenic granuloma or inverted papilloma, leading to delayed diagnosis and suboptimal treatment outcomes.^5^^,^^6^ Histopathological confirmation is essential, as angiosarcoma is characterised by marked endothelial proliferation, irregular vascular channels and high mitotic activity. Immunohistochemical markers such as CD34 and CD31 can aid in distinguishing angiosarcoma from other vascular tumours.^7^ Given its high recurrence rate and metastatic potential, a multimodal treatment approach incorporating surgery, radiotherapy and emerging targeted therapies is typically required for optimal disease control.^8^

This case report highlights diagnostic and therapeutic challenges associated with primary nasal septal angiosarcoma, underscores the necessity for early suspicion and histopathological confirmation and contributes to the ongoing literature review of sinonasal angiosarcoma cases, providing a broader perspective on this rare malignancy.

## Case presentation

A 46-year-old female with no significant medical history presented with intermittent left-sided epistaxis and nasal congestion that persisted for several months. She denied any history of nasal trauma, previous nasal surgery or smoking. Using a nasal endoscope, a 1.0 × 0.5 cm protruding lesion was identified on the left anterior nasal septum (Figure 1).

**Figure 1.**
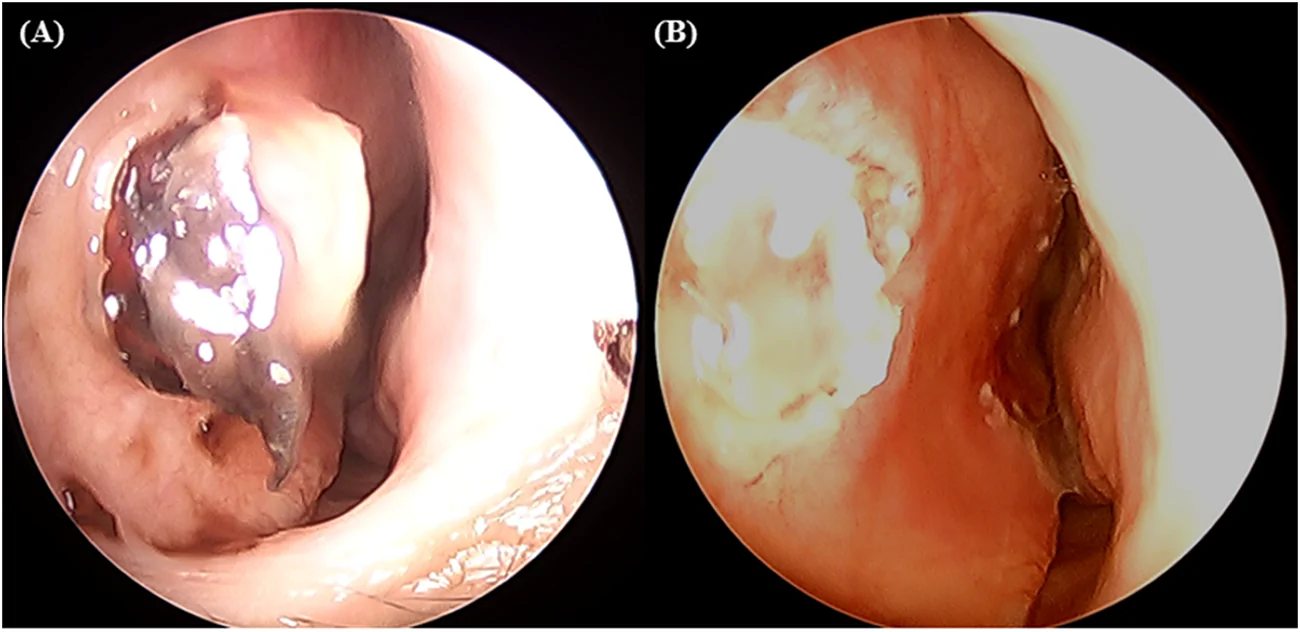
(A) Pre- and (B) post-operative endoscopic photograph of the mass on the left anterior nasal septum.

Routine laboratory investigations including complete blood count and coagulation profile, and inflammatory markers were unremarkable. Pre-operative imaging was not performed because the lesion was small and the patient declined additional imaging. Given the clinical findings, an excisional biopsy was performed under local anaesthesia with 1:100,000 epinephrine infiltration. Intra-operatively, a well-demarcated haemangioma-like mass was identified, without ulceration or necrosis. The septal cartilage and surrounding mucosa appeared intact. The lesion was excised completely on gross inspection and bleeding was successfully controlled.

In Figure 2, histopathological examination revealed proliferating atypical endothelial cells forming irregular vascular channels, with moderate nuclear pleomorphism and a high mitotic rate (24 mitoses per 10 high-power fields). Focal necrosis was observed, accounting for approximately 15 per cent of the tumour area. Although the lesion appeared completely excised macroscopically, histopathology demonstrated diffusely infiltrative margins with residual tumour at the resection edges. Lymphatic, vascular and perineural invasion were absent.

**Figure 2.**
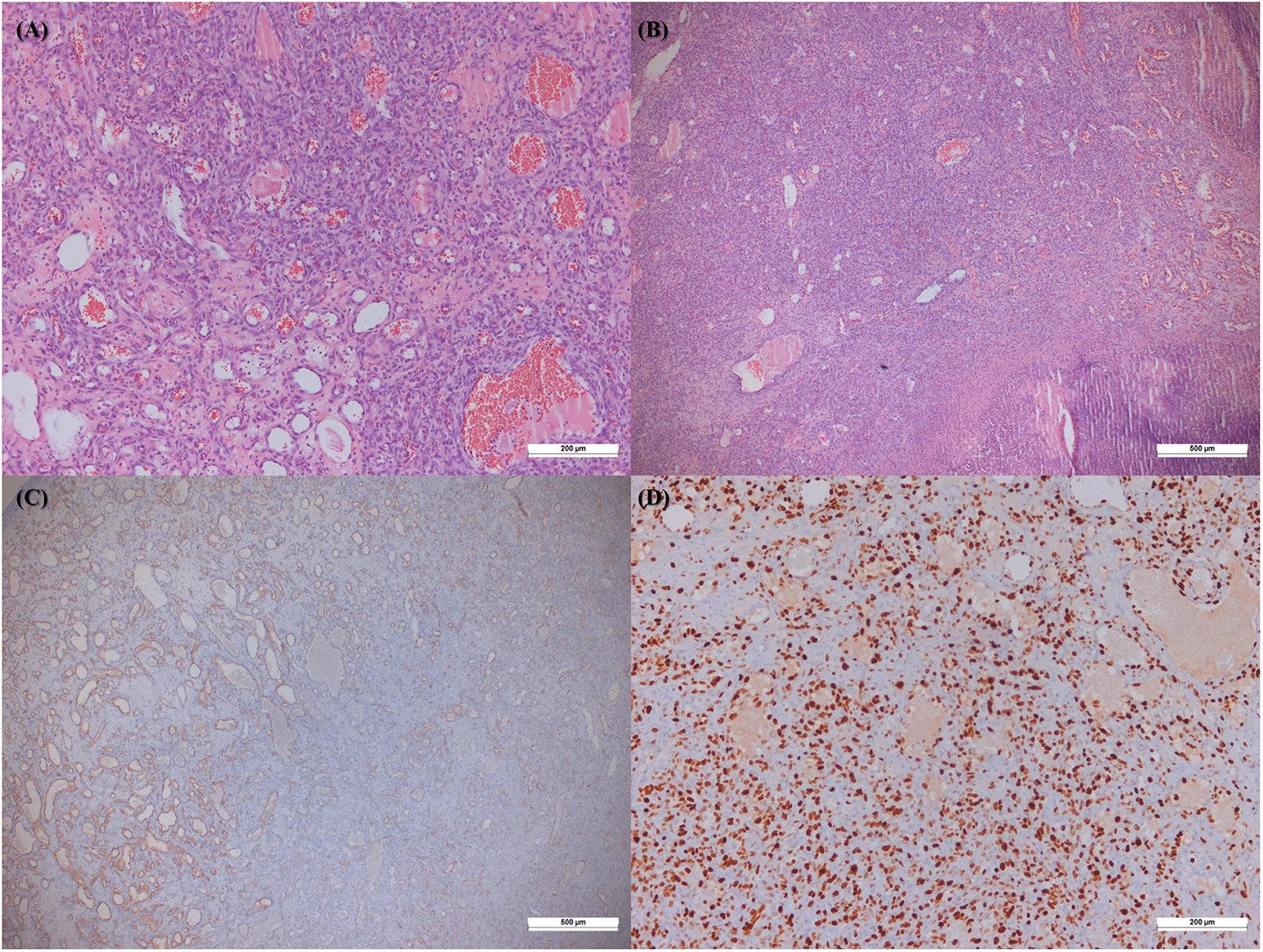
Histopathological features of angiosarcoma. (A) The tumour demonstrates high cellularity with markedly atypical, pleomorphic endothelial cells arranged in solid sheets and irregular vascular channels. (B) Extensive areas of geographic necrosis are observed, indicating aggressive tumour behaviour. (C) Immunohistochemical staining reveals diffuse and strong positivity for CD34, supporting endothelial differentiation. (D) Ki-67 immunostaining highlights a high proliferative index in tumour cells, consistent with the highly proliferative nature of the neoplasm. Figure 2 long description.

Immunohistochemical staining demonstrated strong CD34 positivity, confirming an endothelial origin of the tumour. The Ki-67 proliferation index was approximately 60 per cent, suggesting high proliferative activity. Desmin and actin were negative, ruling out myogenic differentiation. Based on these findings, the patient was referred to an oncology centre at the outside hospital. Post-operative staging with PET-CT performed at an outside hospital showed no regional lymph node or distant metastasis. Given the positive margins and high proliferative index, adjuvant radiotherapy was administered. At 12 months’ follow-up, there was no evidence of local recurrence.

This case report presented the findings derived retrospectively from the patient’s medical records. Therefore, this study did not require an Informed Consent statement, and ethical approval was waived by the Institutional Review Board (exempt number SC25ZISE0029).

## Discussion

Primary angiosarcoma of the nasal septum is an exceptionally rare malignancy, with few cases documented in the literature. Most previously reported cases of sinonasal angiosarcoma involved extensive local invasion, affecting the paranasal sinuses.^6^ Our case was rare in that the angiosarcoma was confined solely to the nasal septum without bony destruction or spread to adjacent tissues, distinguishing it from previously reported sinonasal angiosarcomas.^6^^,^^7^

A review of previously reported sinonasal angiosarcoma cases revealed significant heterogeneity in clinical presentation and prognosis (Table 1). While some patients, such as those reported by Kimura *et al*. and Kurien *et al*., achieved long-term survival following surgical resection.^2^^,^^3^ Others, such as the case reported by Chai *et al*., demonstrated lymphatic metastasis despite no initial distant spread.^6^ These findings emphasise the need for early diagnosis and close follow-up.

**Table 1.** Previously reported sinonasal angiosarcoma Table 1 long description.

Study	Sex/age	Tumour location	Imaging	Pathologic features	Treatment	Follow-up period	Recurrence	Mortality
Panje *et al*., 1986^12^	NA/52	Ethmoid sinus	NA	NA	Surgery	7 years	No	Lymphocytic leukemia
Kurien *et al*., 1989^3^	M/38	Inferior turbinate and middle meatus	Homogeneous opacity of left maxillary antrum with erosion of medial wall	Anastomosing vascular channels, pleomorphic hyperchromatic endothelial cells	Pre-operative radiotherapy and surgery	10 months	No	No
Kimura *et al*., 1992^2^	M/8	Middle turbinate and middle meatus	CT: Opacification of left maxillary antrum, ethmoid and frontal sinus MRI: bright T2 signal	Irregular vascular channels, pleomorphic and hyperchromatic endothelial cells, Factor VIII-positive	Surgery	8 months	No	No
Alameda *et al*., 2000^13^	M/34	Inferior turbinate	Mass extending to the mucosa of inferior turbinate and right maxillary sinus.	Spindle cells around small blood vessels showed mild nuclear pleamorphism and hyperchromasia	Biopsy	9 months	No	No
Treviño-González *et al*., 2009^14^	M/33	Lateral nasal wall	CT: Nonenhanced tumour in the left nostril on the uncinate process.	Vascular neoplasia, composed of multiple blood vessels surrounded by endothelial cells with atypical nuclei	Surgery and post-operative radiotherapy	3 years	No	No
Gravvanis *et al*., 2013^5^	M/68	Nasal cavity, maxillary sinus and septum	CT: Non-enhancing tumour MRI: Tumour invasion of nasal septum, anterior nasal spine and orbicularis oris muscle	Malignant neoplasm of mesenchymal origin, epithelial angiosarcoma, CD31+, CD34+, Ki−67: 35−40%	Surgery and post-operative radiotherapy	29 months	No	No
Es-Sbissi *et al*., 2015^15^	M/53	Middle meatus	CT: heterogeneous contrast enhancement and signs of bone destruction MRI: Low-intensity signal on T1-weighted sequences and a heterogeneous high-intensity signal on T2-weighted sequences with intense, heterogeneous contrast enhancement	Grade II angiosarcoma	Multiple surgery, post-operative chemoradiotherapy and gamma knife radiosurgery	3 years	Yes (5^th^ recurrence)	Yes
Kou and Cheng, 2023^4^	F/70	Maxillary sinus	CT: Soft tissue mass in right maxillary sinus with bone destruction MRI: Isointense/slightly hyperintense on T1, mixed signal on T2	Epithelioid angiosarcoma, irregular vascular spaces, atypical epithelioid cells	NA	NA	NA	NA
Chai *et al*., 2023^6^	M/40	Maxillary sinus and nasal cavity	Space-occupying lesion with bone destruction in the sinus wall and turbinate	CD31+, ERG+, Ki−67 (30%), P16+	Surgery	3 years	Lymphatic metastasis	NA
Present Case	F/46	Nasal septum	Not performed	Irregular vascular channels, pleomorphic endothelial cells, Ki−67: 60%, CD34+, Desmin-	Surgery	Ongoing	NA	NA

CT = computed tomography; MRI = magnetic resonance imaging; NA = not available

Given its nonspecific clinical presentation, nasal septal angiosarcoma is frequently misdiagnosed as a benign vascular lesion, particularly haemangioma, pyogenic granuloma or inverted papilloma. The papillomatous appearance of the lesion in our case raised the possibility of a lobular capillary haemangioma, one of the most common benign vascular tumours in the nasal cavity. However, haemangiomas typically exhibit well-circumscribed margins and lack infiltrative behaviour with a lower mitotic index, which can distinguish them from angiosarcoma.^7^

Due to a high recurrence rate and metastatic potential of nasal septal angiosarcoma, surgical resection alone is often insufficient. While radiotherapy and chemotherapy remain standard adjuvant therapies, recent advances in targeted therapy and immunotherapy offer promising alternatives.^8^

Angiosarcomas are highly vascular tumours, making angiogenesis-related pathways a key therapeutic target. Vascular endothelial growth factor receptor (VEGFR) inhibitors such as bevacizumab have shown limited efficacy as monotherapy. They might provide benefits when combined with paclitaxel. Similarly, pazopanib, a multi-targeted tyrosine kinase inhibitor targeting VEGFR1, VEGFR2 and VEGFR3, has demonstrated moderate clinical benefit, with a response rate of 20 per cent and median progression-free survival of 3 months.^9^

In addition to VEGF-targeted therapies, immune checkpoint inhibitors such as pembrolizumab and ipilimumab have been investigated in angiogenic tumours. One study showed that 30–40 per cent of angiosarcomas expressed PD-L1, suggesting that checkpoint inhibitors could be a viable therapeutic approach for a subset of patients.^10^ While data on angiosarcoma are limited, preliminary studies suggest that ICIs may provide durable responses in tumours with high PD-L1 expression.^10^ A phase II clinical trial is currently investigating avelumab combined with paclitaxel for locally advanced and metastatic angiosarcoma.^10^

Another emerging target is the PI3K/AKT/mTOR signalling pathway, which plays a crucial role in tumour proliferation and angiogenesis. mTOR inhibitors such as everolimus and temsirolimus have demonstrated an ability to inhibit tumour growth in preclinical models, although their clinical efficacy remains under investigation.^8^ Combination therapy using mTOR and MEK inhibitors has shown promising results, significantly reducing tumour growth in angiosarcoma cell models.^8^ In addition, recent genomic studies have identified recurrent mutations in PIK3CA and PLCG1, suggesting that PI3K pathway inhibitors could be a potential therapeutic approach.^11^ Some angiosarcomas also exhibit RAS/RAF/MEK pathway alterations, making MEK inhibitors another possible treatment strategy.^11^ However, clinical responses to these inhibitors have been variable, highlighting the need for biomarker-driven treatment selection.^11^

Despite these promising developments, angiosarcoma remains a challenging malignancy to treat, showing variable response rates to targeted therapies. Further research is needed to identify biomarkers that can predict treatment response and to develop more effective therapeutic strategies.^11^

A key limitation of this case report is the lack of pre-operative cross-sectional imaging (computed tomography [CT] and/or magnetic resonance imaging [MRI]), which is widely regarded as standard practice prior to biopsy or excision of sinonasal vascular lesions. In this case, imaging was not obtained because the lesion was small and the patient declined additional investigations. To mitigate this limitation, post-operative staging with positron emission tomography (PET)-CT demonstrated no regional lymph node or distant metastasis, and the patient received adjuvant radiotherapy owing to positive margins. She remains disease-free at 12 months.

## Conclusion

We present an exceptionally rare case of primary angiosarcoma confined to the nasal septum with no evidence of adjacent tissue invasion or identifiable predisposing factors. This report highlights the importance of maintaining a high index of suspicion for patients presenting with recurrent epistaxis and nasal congestion, given the potential for misdiagnosis as a benign vascular lesion. Due to the aggressive nature of angiosarcoma, a multidisciplinary approach incorporating surgery and radiotherapy is often required, with systemic therapies considered for optimising patient outcomes. Prospective data remain limited in sinonasal angiosarcoma, and collaborative registries may help define optimal multimodal strategies.

## Editorial assistance

The English in this document has been checked by at least two professional editors, both native speakers of English. For a certificate, please see: https://www.harrisco.net/mypage/cert_down.php?od_srl=237365&v=e9733ac62a75d06c33776c90bc02df46

## Figure 2 Long description

The image A showing a purple and pink stained tissue section with many small dark-purple nuclei, multiple irregular pale spaces and several red areas. A white scale bar is present at the lower right. The image B showing a purple and pink stained tissue section with a large pale region on the right and denser purple tissue on the left. Several red areas are scattered. A white scale bar is present at the lower right. The image C showing a pale gray background with a light brown branching network pattern across the field. A white scale bar is present at the lower right. The image D showing a pale background with numerous small brown stained dots distributed across the field, with lighter open spaces between clusters. A white scale bar is present at the lower right.

Navigate back to Figure 2.

## Table 1 Long description

The table summarizes 10 published cases plus a current case of sinonasal angiosarcoma, listing patient sex and age, tumour site, imaging findings, pathology, treatment, follow-up, recurrence, and mortality. Ages range from 8 to 70 years, with most patients male; the current case is a 46-year-old female with a nasal septum tumour. Tumours most often involve the maxillary sinus, nasal cavity, turbinates, or middle meatus, with imaging commonly describing a mass and, in several cases, bone erosion or destruction. Pathology descriptions frequently note irregular or anastomosing vascular channels with pleomorphic, hyperchromatic endothelial cells, and several reports include endothelial markers such as CD31, CD34, ERG, or Factor VIII; reported proliferation indices vary, including about 30 to 40 percent and 60 percent in the current case. Treatment is usually surgery alone or surgery with post-operative radiotherapy; one case received multiple surgeries plus combined chemo-radiotherapy and radiosurgery. Follow-up ranges from 8 months to 7 years when reported, and most cases report no recurrence. Notable exceptions include one patient with a fifth recurrence who died, and another with lymphatic metastasis at 3 years; several recent reports have missing outcome data, limiting comparisons.

Navigate back to Table 1.
